# The role of artificial intelligence based on PET/CT radiomics in NSCLC: Disease management, opportunities, and challenges

**DOI:** 10.3389/fonc.2023.1133164

**Published:** 2023-03-07

**Authors:** Qiuyuan Hu, Ke Li, Conghui Yang, Yue Wang, Rong Huang, Mingqiu Gu, Yuqiang Xiao, Yunchao Huang, Long Chen

**Affiliations:** ^1^ Department of positron emission tomography/computed tomography (PET/CT) Center, Yunnan Cancer Hospital, The Third Affiliated Hospital of Kunming Medical University, Cancer Center of Yunnan Province, Kunming, Yunnan, China; ^2^ Department of Cancer Biotherapy Center, Yunnan Cancer Hospital, The Third Affiliated Hospital of Kunming Medical University, Cancer Center of Yunnan Province, Kunming, Yunnan, China; ^3^ Department of Thoracic Surgery I, Key Laboratory of Lung Cancer of Yunnan Province, Yunnan Cancer Hospital, The Third Affiliated Hospital of Kunming Medical University, Cancer Center of Yunnan Province, Kunming, Yunnan, China

**Keywords:** PET/CT, NSCLC, radiomics, artificial intelligence, lung cancer

## Abstract

**Objectives:**

Lung cancer has been widely characterized through radiomics and artificial intelligence (AI). This review aims to summarize the published studies of AI based on positron emission tomography/computed tomography (PET/CT) radiomics in non-small-cell lung cancer (NSCLC).

**Materials and methods:**

A comprehensive search of literature published between 2012 and 2022 was conducted on the PubMed database. There were no language or publication status restrictions on the search. About 127 articles in the search results were screened and gradually excluded according to the exclusion criteria. Finally, this review included 39 articles for analysis.

**Results:**

Classification is conducted according to purposes and several studies were identified at each stage of disease:1) Cancer detection (n=8), 2) histology and stage of cancer (n=11), 3) metastases (n=6), 4) genotype (n=6), 5) treatment outcome and survival (n=8). There is a wide range of heterogeneity among studies due to differences in patient sources, evaluation criteria and workflow of radiomics. On the whole, most models show diagnostic performance comparable to or even better than experts, and the common problems are repeatability and clinical transformability.

**Conclusion:**

AI-based PET/CT Radiomics play potential roles in NSCLC clinical management. However, there is still a long way to go before being translated into clinical application. Large-scale, multi-center, prospective research is the direction of future efforts, while we need to face the risk of repeatability of radiomics features and the limitation of access to large databases.

## Introduction

Lung cancer is one of the most common malignant tumors in the world and the leading cause of cancer-related death, with a five-year survival rate of 21.7% (1). Although this figure is higher than before, lung cancer is still a primary disease threatening human health. Non–small cell lung carcinoma (NSCLC) is the most common type of lung cancer, including adenocarcinoma, squamous cell carcinoma, adenosquamous carcinoma, large cell carcinoma, and sarcomatoid carcinoma (2). Tumor progression, prognosis evaluation, and determination of treatment plans are mainly dependent on the stage of the tumor and the histopathologic subtype. 18F-fluorodeoxyglucose positron emission tomography/computed tomography (18F-FDG PET/CT) is a fusion imaging technique that can reflect the anatomical structure and functional metabolic information of the lesions simultaneously. It has become one of the popular diagnostic tools in oncology due to abundant imaging information and high sensitivity it can provide. The guidelines of the National Comprehensive Cancer Network (NCCN) recommend 18F-FDG PET/CT for evaluation of patients from stage I to stage IV NSCLC and an incidentally detected lung nodule measuring more than 8 mm (3). In addition, 18F-FDG PET/CT also shows unique value in the prognosis and therapeutic response assessment (4–6).

Personalized medicine is the goal of modern cancer treatment, which aims to link genomic and clinical profiles of individual patients for more targeted therapies of tumors. Now, we recognize lung cancer as a molecularly heterogeneous disease and believe that understanding the link between underlying biology and clinical behavior is crucial for therapeutic decision-making (7). There is evidence that medical images can reflect the heterogeneity of tumors, such as cellular metabolism, necrosis, cancer-associated fibroblast, and expression of specific receptors (8, 9). Elevated glucose uptake is a hallmark of cancer. According to previous studies, tumor size, histological subtypes, and prognosis of NSCLC had influences on fluorodeoxyglucose (FDG) uptake (10–12). The standardized uptake value (SUV) is a semi-quantitative index reflecting FDG uptake in PET/CT, and the higher SUV was believed to indicate the biological aggressiveness of tumors (13). However, it does not always accurately convey tumor responses. A positive PET finding can be caused by infection or inflammation (14), while false-negative findings can result from some small nodules, low cellular density in lesions such as ground-glass opacity, or low tumor avidity of FDG (15).In the past decade, it has been recognized that medical images contain more helpful information that can not be captured by the naked eye but can be obtained by computer extraction and analysis, leading to better disease management.

**Figure 1:**
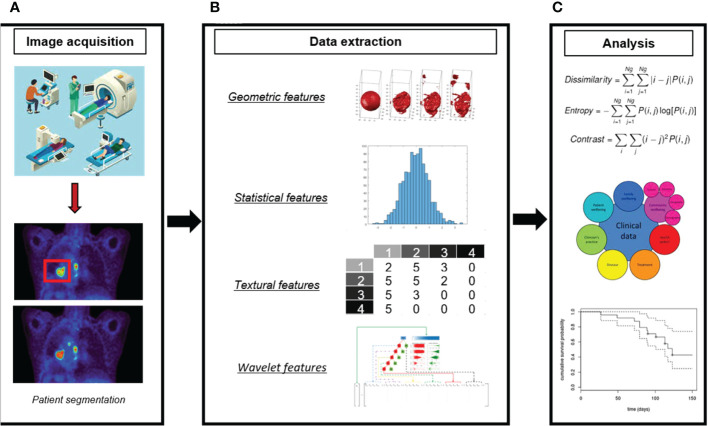
A workflow of radiomics analysis (18). **(A)** Image acquisition and ROI segmentation. **(B)** Extraction of radiomic features. **(C)** Model development.

The introduction of radiomics analysis provides a promising new approach to cancer assessment. Radiomics is the application of an automatic data characterization algorithm to transform image data of the region of interest (ROI) into high-dimensional feature data that can be mined (16). In radiomics analysis, the image information is quantified for analysis by the computer, which strengthens the traditional manual diagnosis. A complete radiomics analysis pipeline involves many steps (17): i) identifying the clinical question and target patient cohorts, ii) determining the imaging mode for radiomics analysis, iii)standardizing image acquisition and reconstruction, iv) segmenting tumor and defining ROI, v) extracting and selecting features, vi) building models with different machine learning algorithms, vii)training and validating the radiomics model. **Figure 1** displays a workflow of radiomics analysis (18). To facilitate the clinical translation of radiomics, it is necessary to improve the robustness and repeatability of imaging markers. So far, many researchers have made efforts to this end. A set of 169 radiomics features was standardized by the Image Biomarker Standardization Initiative (IBSI) (19) so that different radiomics software can be verified and calibrated.

The term “artificial intelligence” was coined by John McCarthy in 1955, defined as “the science and engineering of making intelligent machines” (20). Today, AI is developing rapidly in many fields, and the definitions in different kinds of literature are abundant and confusing. The AI mentioned in this review specifically refers to the virtual branch of medicine (20), that is, AI technology represented by machine learning (ML) and deep learning (DL). According to their purposes and how the underlying machine is taught, ML algorithms can be divided into three categories: supervised, unsupervised and semi-supervised (21). Currently, most ML algorithms under the background of predictive oncology are based on supervised learning. Several classical ML algorithms include random forest (RF), Support Vector Machine (SVM), Decision Trees, Naïve Bayes, and k-nearest Neighbors (22). As the state-of-the-art subcategory of ML, deep learning is essentially a variant of the artificial neural network. In deep learning, the research of the Convolution neural network(CNN) (23) has attracted much attention. CNN is a kind of multi-layer feed-forward neural network, while low-level image features are extracted by early hidden layers, and progressively higher features are learned by successive layers before classification by different classifiers. Programs have been created to automatically perform all radiomics analysis tasks from tumor segmentation to model building using AI (24–27). At present, radiomics-based ML models (or DL models) are widely developed for whole-process management of lung cancer: diagnosis, staging, treatment planning, and prognosis, so on. Some results have reported superior performance compared to traditional statistical methods or even better than radiologists.

The primary purpose of this review is to sort out and summarize the application of 18F-FDG PET/CT radiomics combined with artificial intelligence in the disease management of NSCLC, as well as opportunities and challenges.

## Materials and methods

**Figure 2:**
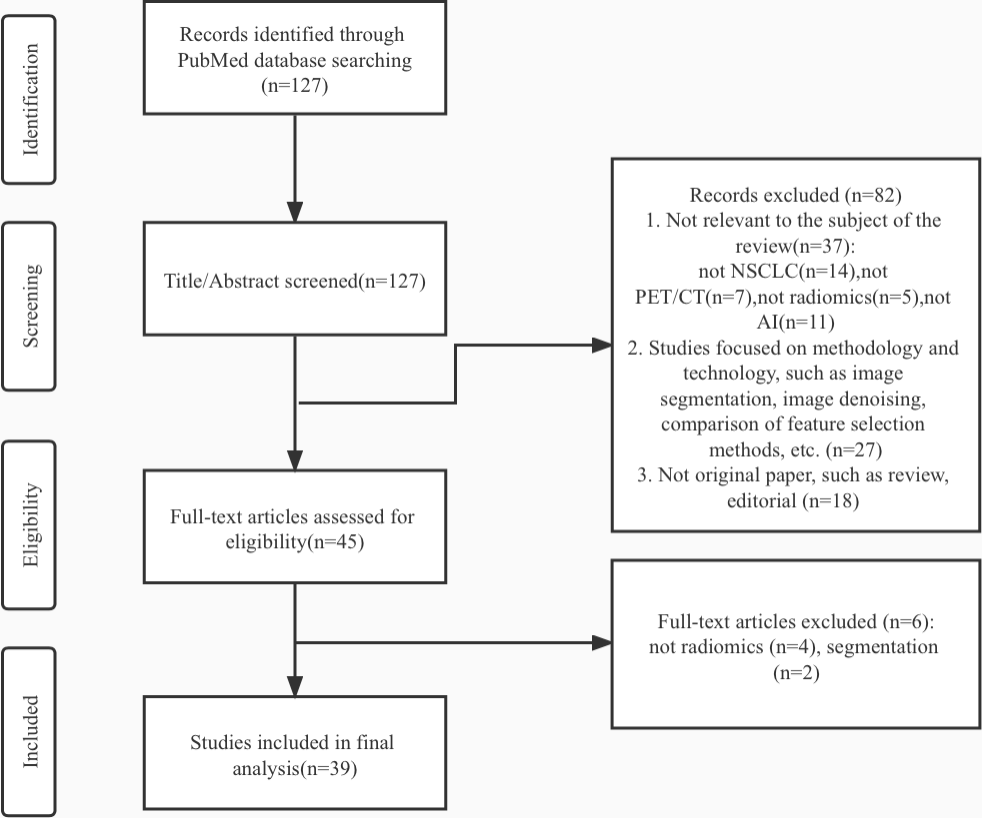
Flow chart of the study selection process.

A comprehensive search of papers published from 2012 to 2022 was conducted on the PubMed database. The following keywords and Medical Subject Heading (MeSH) words are used to generate different searches: “Carcinoma, Non-Small-Cell Lung”[Mesh], “lung neoplasm” [Mesh], “lung cancer”, “Positron Emission Tomography Computed Tomography”[Mesh], “Positron-Emission Tomography”[Mesh], “radiomic*”, “Artificial Intelligence”[Mesh], “Machine Learning”[Mesh], “Deep Learning” [Mesh]. We applied the following exclusion criteria: (1) Studies not aimed at assisting clinical management decision-making of NSCLC, such as screening, diagnosing, characterizing, predicting outcomes, or evaluating prognosis;(2)Studies not combinate PET/CT radiomics and artificial intelligence simultaneously;(3) Studies focused on methodology and technology of radiomics or AI, such as image segmentation, image denoising, comparison of feature selection methods; (4) Articles without original data, such as reviews and editorials. No study was excluded due to language and geographical location. About 127 articles in the search results were screened and gradually excluded according to the criteria in **Figure 2**. Then, this review included 39 articles for final analysis. We identified several articles at every step of disease management: Cancer detection(n=8), histology and stage of cancer(n=11), metastases(n=6), genotype(n=6), treatment outcome, and survival(n=8).

## Results

### Cancer detection and characterization

Lung cancer often occurs in the form of pulmonary nodules in the early stage, with a diameter of no more than 3 cm (28). Although the wide application of thin-layer CT has greatly improved the detection rate of pulmonary nodules, accurate localization and characterization is still a difficult problem for radiologists, especially when the nodules are very small. Compared with CT imaging, PET/CT shows a stronger ability to detect solitary pulmonary nodules. One large cohort study of patients with tumors reported that FDG-negative sub-centimeter pulmonary nodules are benign in 98% (29). It is recognized that integrating AI into radiomics can further reduce the false positive rate of diagnosis (30, 31). Traditional AI-assisted diagnosis is only based on CT features, so it is possible to misdiagnose vascular or non-nodular areas as solitary pulmonary nodules, resulting in a high false positive rate. Based on the ability of PET imaging to extract phenotypic and functional tumor heterogeneity information, predictive models have been established to extract quantitative features from PET/CT to distinguish benign and malignant nodules. Almost all studies achieved the best performance of the model by combining PET and CT features. Most prediction models for differentiating benign and malignant pulmonary nodules are developed based on machine learning algorithms of SVM. Support vector machine (SVM) is a generalized linear binary classifier through supervised learning, building the decision function based on a specific kernel function and the penalty parameter to avoid over-fitting. It is especially suitable for the case of a limited sample size. In a retrospective analysis, after adding delayed PET features, the SVM model shows higher diagnostic accuracy than the physician evaluation and common clinical indicators (32).

The deep learning network shows excellent performance in the prediction model. A study found that the artificial neural network showed excellent predictive value in estimating the likelihood of malignancy (AUC 0.981). The sensitivity and specificity corresponding to the optimal critical point of the ROC curve were 100% and 93.1%, respectively (33). Some studies try to realize automation from the definition of regions of interest, a high-resolution network (34) is proposed to automatically detect and classify pulmonary nodules without any stride or pooling to retain the high-resolution imaging features.

Not only lung cancer but also some benign diseases have high FDG uptake, such as granulomatous disease and inflammatory pseudotumor. Zhang et al. (35) reported that the SurfaceVolumeRatio of the CT-radiomics features and SUVpeak of the PET metabolic parameters have significance in differentiating benign and malignant lung lesions. And SurfaceVolumeRatio has a unique ability to distinguish granulomatous lesions from inflammatory pseudotumors. Sarcoidosis and lymphoma often present similar features on PET/CT, such as severely swollen lymph nodes and hypermetabolic changes involving multiple systems throughout the body. A study differentiates sarcoidosis and lymphomas with seven different feature selection approaches and four different ML classifiers. At the lesion level, they found highly accurate signatures to create models to differentiate cancer vs. sarcoidosis (AUC 0.94) and Hodgkin vs. diffuse large B-cell lymphoma (AUC 0.95) (36). In a more extensive cohort retrospective study (37), the researchers used a CNN to automatically locate and classify PET uptake patterns in lung cancer and lymphoma. It is confirmed that the addition of the atlas information (AUC 0.99) was the best feature combination, which performed better compared with the PET/CT with maximum intensity projection combination (P <0.001). We summarized the main findings on lung nodule prediction of malignancy in **Table 1**.

**Table 1:** Main findings on lung nodule prediction of malignancy.

Aim	Reference	Studies type	Data set	Number of features	Model	Validation	External testing	Results
Detection of pulmonary nodules	Zhao et al., 2015 (31)	Single center, retrospective analysis	219	6	SVM	Ten-fold cross validation	None	sensitivity 0.956
	Scott et al., 2019 (33)	Single center, retrospective analysis	125	6	ANNs	Split sample (40)	None	AUC 0.98
	Chen et al., 2017 (32)	Single center, retrospective analysis	85	Unknown	SVM	Five-fold cross validation	None	AUC 0.91
Distinction of lymphoma from sarcoidosis	Lovinfosse, et al., 2022 (36)	Single center, retrospective analysis	420	8	ML(RF,SVM,NB, logistic regression)	Five-fold cross validation	None	RF model (AUC 0.94)
Localization and uptake classification of lung cancer and lymphoma	Sibille et al., 2020 (37)	Single center, retrospective analysis	629	5	CNN	Split sample	None	AUC 0.988
Differentiation of benign and malignant lung lesions	Zhang et al., 2019 (35)	Single center, retrospective analysis	135	17	SVM	Five-fold cross validation	None	AUC 0.887

NB, naive bayes; RF, random forest; SVM, support vector machine.

### Histology and staging

NSCLC is a group of heterogeneous diseases, of which lung adenocarcinoma and lung squamous cell carcinoma are the most common subtypes. Different subtypes of NSCLC have different phenotypic and biological characteristics, leading to significant differences in disease progression and prognosis (7). Hence, it is necessary to make different treatment plans for different subtypes.

The gold standard of histological classification is pathological evaluation. At present, many techniques can be used for tissue diagnosis and the preferred choice is image-guided needle core biopsy (38). Unfortunately, as an invasive examination, the operational risks of biopsy are inevitable, including a series of complications such as infection. Moreover, the spatio-temporal pathological heterogeneity of tumors limits the ability to capture biodiversity or disease evolution in a single biopsy (39). By contrast, the extraction of quantitative radiological features from non-invasive imaging can reflect a wealth of information about genotype, tumor microenvironment, and sensitivity to treatment (17, 40). Unlike most previous studies that explored only one model, recent studies compared the combination of different feature selection methods and machine learning algorithms to build models and evaluate the best performance (41). The RF model and SVM model performed well in many studies, and deep learning is superior to all traditional machine learning. Han et al. extracted 688 features from each ROI, and finally selected the top 50 features of each feature selection method for analysis. The best performance of subtype classification was achieved when linear discriminant analysis (AUC 0.863) and SVM (AUC 0.863) classifiers were combined with the L2,1NR feature selection method. In addition, they evaluated the VGG16 deep learning model based on transfer learning, outperforming all traditional machine learning algorithms (AUC 0.903) (42). Zhou et al. evaluated five feature selection methods and nine classification methods, the gradient boosting decision tree and the RF was considered optimal classification methods for the PET and CT datasets, respectively (43). Some studies have shown that the ability of the model to distinguish histological subtypes can be further improved by combining clinical features and tumor markers (44). Zhao et al. use the Boruta algorithm to determine an optimal subset of 13 features, including two clinical features(sex and smoking history), two laboratory indicators(CEA and SCCA), and nine PEF/CT radiomic features (38). The clinical features they included were the same as those of Ren et al (44), but there were differences in laboratory indicators. Ren et al. proposed that SCCA and CYFRA21-1 were statistically significant, while CEA levels were not statistically different between adenocarcinoma and squamous cell carcinoma patients.

The TNM staging system is a standard tool for evaluating the primary tumor, regional lymph node metastasis (LNM), and distant metastasis (45). In previous work, the CNN algorithm was developed and tested to distinguish T1-T2 from T3-T4 lung cancer on PET/CT images, achieving 69% accuracy, 70% recall and 67% specificity in the test set (46). Recently, Kasinathan et al. proposed a Cloud-based Lung Tumor Detector and Stage Classifier based on PET/CT images. The model achieved an average classification accuracy of 99.1% and an average accuracy of 98.6% (47). We summarized the main findings on histology and staging in **Table 2**.

**Table 2:** Main findings on histology and staging.

Aim	Reference	Studies type	Data set	Number of features	Model	Validation	External testing	Results
Histology	Han et al., 2021 (42)	Single center, retrospective analysis	1419	50	10 ML classifiers and 1 DL classifier (VGG16)	Ten-fold cross validation	None	**V**GG16 (AUC 0.903; accuracy 0.841)
	Zhou et al., 2021 (43)	Single center, retrospective analysis	769	Unknown	9 ML classifiers	Ten-fold cross validation	None	GBDT-GBDT (AUC 0.897) GBDT-RF (AUC 0.839)
	Zhao et al., 2022 (38)	Single center, retrospective analysis	120	13	10 ML algorithms	Split sample	None	SVM model (AUC 0.876) RF model (AUC 0.863)
	Hyun et al., 2019 (41)	Single center, retrospective analysis	396	44	5 ML algorithms	Random sampling	None	LR model (AUC 0.859)
Staging	Kirienko et al., 2018 (46)	Single center, retrospective analysis	472	Unknown	CNN	Five-fold cross validation	None	AUC 0.68; accuracy 0.69
	Kasinathan et al., 2022 (47)	Single center, retrospective analysis	94	Unknown	Cloud-LTDSC	Ten-fold cross validation	None	accuracy 0.97

LR, logistic regression; GBDT, gradient boosting decision tree.

### Metastases

NSCLC is usually accompanied by metastasis of mediastinal and hilar lymph nodes, and the identification of LNM significantly affects the staging, prognosis and treatment. Yin et al. developed a classification method based on SVM to improve the diagnostic performance of LNM. They converted the predictive model including the optimal feature SURblood into scoring rules to help clinicians make decisions (48). To solve the time-consuming problem caused by manual localization of nodules in previous studies, Wallis et al. used a U-Net to automatically identify candidate regions on PET/CT images and a 3D CNN model to classify regional lymph nodes. Through further fine-tuning the model using transfer learning, their model achieved good performance on test sets of different scanners (49).

Furthermore, there is also great clinical significance in the distinction of second primary lung cancers from pulmonary metastases because of the vastly different survival outcomes between them (50). D’Arnese et al. proposed LuCIFEx, a fully automated three-stage pipeline for the characterization of NSCLC. Similarly, LuCIFEx pipeline automatically segmented lesions on PET/CT images without manual annotation, and output the results through RF classification method after calculating the accurate radiation features. The accuracy of their model in distinguishing primary NSCLC from lung metastases is 92.3 ± 1.9% (51).

### Genotype

Over the past two decades, the emergence of various targeted therapies and immunotherapies has provided more individualized treatment decisions for patients with advanced NSCLC. The diagnosis of tumors at the molecular level is crucial for the accurate determination of targeted therapy. Different driver mutations have been identified in NSCLC, including oncogenic mutations in epidermal growth factor receptor (EGFR), anaplastic lymphoma kinase (ALK), BRAF, ROS1, and MET (52). If feasible, NCCN recommends that eligible patients with metastatic NSCLC be tested for molecular and biomarkers of disease-related mutations before starting treatment (1).

Early identification of EGFR mutational status is important before tyrosine kinase inhibitors treatment. There have been conflicting results that 18F-FDG uptakes may not be a reliable marker for predicting EGFR mutation status (53, 54). Present studies have validated that it is more convincing to use some PET/CT radiological features to predict EGFR mutation state (55, 56). In one large multi-center study (57), a small-residual-convolutional-network model was proposed to distinguish EGFR-mutant type from wild type, and the deep learning score (DLS) performed well with the AUC of 0.81 in the external test cohort. There was apparent heterogeneity in clinical characteristics and image acquisition because the patient cohorts came from different institutions. However, this could be regarded as an advantage, because this heterogeneity can avoid the possibility of over-fitting caused by a specific patient cohort or imaging parameters to some extent, resulting in a more robust and portable model. A similar DLS was also used to predict programmed death-ligand 1(PD-L1) status, which is the checkpoint target determined by immunohistochemistry. It was shown that the PD-L1 DLS significantly discriminated between PD-L1 positive and negative patients (AUC≥0.82 in the training, validation, and two external test cohorts) (58).

Identifying EFGR mutation subtypes is essential to guide more accurate personalized treatment. Increasing evidence has demonstrated that patients with E19 del mutation have a higher response to EGFR tyrosine kinase inhibitors (TKIs) treatment than those with E21 mis mutation, and have longer survival time (59). Liu et al. extracted and selected two sets of PET/CT radiomic features to identify E19 del mutation or E21 mis mutation, the AUC of radiomics features of the two groups is 0.77 and 0.92 respectively (60). The main clinical tools for detecting ALK rearrangement include immunohistochemistry and fluorescence in situ hybridization. Chang et al (61). firstly proposed a novel ML model to predict the ALK mutation status based on PET/CT images. They also confirmed that age and pleural effusion were independent predictors. The PET/CT-clinical combined model has a significant advantage in predicting the ALK mutation status, with the highest AUC value (0.88) in the test group. In addition, another study has successfully predicted the tumor immune microenvironment phenotype of NSCLC based on the PET/CT-clinical combined model (62). We summarized the main findings on genotype in **Table 3**.

**Table 3:** Main findings on genotype.

Aim	Reference	Studies type	Data set	Number of features	Model	Validation	External testing	Results
EFGR mutation status, subtypes	Liu et al., 2020 (60)	Multi-center, retrospective analysis	148	10	XgBoost	3-fold cross validation	None	**g**eneral EGFR mutation (AUC 0.87) E19 del mutation (AUC 0.77) E21 mis mutation (AUC 0.92)
	Nair et al., 2021 (56)	Single center, retrospective analysis	50	14	LR	LOOCV*	None	AUC 0.87
	Mu et al., 2020 (57)	Multi-center, retrospective analysis	616	Deeply learned features(N=256)	SResCNN	Split sample	Yes	AUC 0.81
Measurement of PD-L1 status	Mu et al., 2020 (58)	Multi-center, retrospective analysis	697	Deeply learned features(N=256)	SResCNN	Split sample	Yes	AUC 0.82
Predicting TIME profiles	Tong et al., 2022 (62)	Single center, retrospective analysis	221+39	9	Multivariate regression	Split sample	Yes	AUC 0.932
Predicting ALK rearrangement status	Chang et al., 2021 (61)	Single center, retrospective analysis	526	25	LASSO	100-folds LGOCV	None	AUC 0.88

ALK, Anaplastic lymphoma kinase; LOOCV, the leave-one-out cross-validation; LASSO, Least absolute shrinkage and selection operator; LGOCV, leave-group-out cross-validation; SResCNN, small-residual-convolutional-network; TIME, The tumor immune microenvironment.

### Treatment outcome and survival

One goal of radiomics is to construct models for predicting treatment response and prognosis from medical images in the context of tumor characteristics. Radiological criteria are usually used as a substitute for pathology to evaluate the response of tumor treatment, including the definitions of complete response, partial response, stable disease, and progressive disease (63). The evaluation of the efficacy of World Health Organization (WHO) criteria or Response Evaluation Criteria in Solid Tumors (RECIST) was entirely based on the anatomical size of the tumor. Since nuclear medical imaging can provide information on metabolic activity other than anatomical imaging, PET/CT is particularly valuable in evaluating tumor treatment response (64). PET Response Criteria in Solid Tumors (PERCIST 1.0) provides a systematic and structured method for assessing response to therapy in cancer patients with 18F-FDG PET (65).

In the field of radiomics, several studies have been published to evaluate the response to radiotherapy or immunotherapy based on ML algorithms, but most of them are based on CT images (66). Yoo et al. firstly constructed an RF model by extracting texture features from PET/CT images to assess the neoadjuvant concurrent chemoradiotherapy (CCRT) response of patients with stage III NSCLC. The pathological complete response after neoadjuvant CCRT is an independent prognostic factor for recurrence-free survival (RFS), and overall survival (OS) in NSCLC (67). In comparison with PET parameters and physician reporting, the ML model had a significantly higher accuracy of 93.4% (p < 0.05) (68).

Stereotactic body radiotherapy (SBRT) is now a standard of care option for patients with NSCLC, especially those not candidates for surgery. In some previous studies, the radiomics features used to build models differed significantly, which may be attributed to different patient cohorts, scanners, and segmentation/intensity discretization schemes (69). These radiomics features that were not in conformity with the IBSI standardization framework were not repetitive and comparative. It was demonstrated that the ComBat harmonization method (70) could coordinate the radiomics features derived from different acquisition and reconstruction parameters, including PET, CT and MRI imaging of NSCLC, nervous system and cervical cancer (71). Dissaux et al. applied this algorithm to a multi-center study, and by combining one feature from PET, and one from CT, the best-performing model was obtained, reaching a sensitivity of 100% and a specificity of 96% (72).

The European Society for Medical Oncology (EMSO) recommends that NSCLC patients treated with radical intent should be followed for treatment-related complications and detection of treatable relapse (73). Ahn et al. established ML models to improve clinical recurrence risk stratification, and the contrast and busyness texture features from neighborhood grey-level difference matrix were proved to be the best two predictors (74). A multi-center study compared multiple feature selection methods and ML classification algorithms, constructing a risk-stratification model for predicting recurrence, RFS and OS (75). Their model showed consistency across validation and external test sets and provide more accurate results than traditional Cox proportional-hazards models. Another multi-center study explored the relationship between tumor immune status and RFS and OS in patients receiving immunotherapy through deep learning (76). Most of the current work is based on RFS and OS, the two leading indicators to be output as the results of prediction models. Although these studies have achieved varied success, they have focused on predicting the binary results at specific time points. Huang et al. extend features extracted with the CNN to the prediction of survival as a continuous outcome by incorporating the random survival forest model, which outperformed corresponding CT-only and PET-only models (77). We summarized the main findings on treatment outcome and survival in **Table 4**.

**Table 4:** Main findings on treatment outcome and survival.

Aim	Reference	Studies type	Data set	Number of features	Model	Validation	External testing	Results
Treatment Response to CRRT	You et al., 2022 (68)	Single center, retrospective analysis	430	30	RF	10-fold cross validation	None	accuracy 0.934
Local control after SBRT	Dissaux et al., 2020 (72)	Multi-center, retrospective analysis	87	2	Multivariate regression	Split sample	Yes	sensitivity 1.0 specificity 0.96
Response to immunotherapy	Park et al., 2020 (76)	Multi-center, retrospective analysis	152	Deeply learned features	3D CNN	10-fold cross validation	Yes	Spearman rho -0.54
Survival Predicting recurrence and survival	Hindocha et al., 2022 (75)	Multi-center, retrospective analysis	657	34	10 ML algorithms	10-fold cross validation	Yes	RFS (AUC 0.681) Recurrence (AUC 0.722) OS (AUC 0.717)
Predicting the recurrence risk	Ahh et al., 2019 (74)	Single center, retrospective analysis	93	35	5 ML algorithms	Split sample	None	RF model (AUC 0.956, accuracy 0.901)
Predicting progression and OS	Huang et al., 2022 (77)	Multi-center, retrospective analysis	1168	16	CNN, RF	Split sample	None	progression (AUC=0.876, accuracy=0.790) OS (C-index=0.737)

CRRT, concurrent chemoradiotherapy; OS, overall survival; RFS, recurrence-free survival; SBRT, stereotactic body radiotherapy.

## Discussion

Radiomics has sprung up rapidly in the past decade, and developing reliable computer-aided diagnosis systems based on AI has been recognized as an important research field in medical imaging. Although radiology has been used in many diseases, lung cancer is the most widely studied and characterized malignant tumor so far (78). This review aims to introduce and summarize the published literature on the subject of PET/CT radiomics combined with AI in the field of NSCLC, including tumor detection, histology, staging, treatment response assessment, prognosis prediction, and prediction of recurrence and metastasis. An exciting news is that most of the models developed in these works performed well, with predictive performance comparable to or even better than that of human experts, which confirms that the hope is feasible to apply AI to medical images to assist radiologists in improving diagnostic accuracy and efficiency.

However, none of them has been applied to a certain scale of clinical trials, which means there is little high-quality evidence that the work of clinicians or the disease outcomes of patients have been improved.

The process of model building, training, verification, and testing is essential before moving on a technology to a clinical trial. After training a ML or DL model, a validation set is generally used for guiding the optimization of the parameters. The cross-validation method and random sampling are most commonly used to separate validation sets. Previous studies have reported that the performance of the training set and validation set of classifiers is generally optimistically biased under a limited sample size (79). To evaluate the real performance of the model in unknown situations, an independent test set is necessary, which was not learned by the model during the training process, preferably external (80). However, most of the published studies so far only contain cross-validation results, that is, confusing the meaning of verification set and test set.

Overviewing these works, we only found that one prospective study cohort was used as an external test cohort to test the model by Mu et al. (57), while the rest of the studies are all retrospective. One of the major reasons may be the long time-taking and great difficulty of prospective study design.

A large enough training sample size is crucial for developing a robust machine algorithm, especially deep learning depends on a large number of data sets (81). However, the available clinical data is limited. Even though there are some published multi-institution cohort studies, achieving a smooth flow of data among a wide range of institutions is difficult. An effective method used by deep learning developers to solve limited samples is transfer learning, that is, fine-tuning CNN models pre-trained from natural image datasets to medical image tasks. It is reported that transfer learning was generally beneficial in improving the training convergence and robustness of the CNN model (82).

Although we emphasize the need for multi-center research, there is a repeatable risk problem for radiology. For example, different scanning protocols and image reconstruction methods may lead to limited performance or result deviation of radiomics models. Selection of reproducible and robust features is an arduous and prudent work, and there is great heterogeneity between the radiological features extracted by the current work. Many variables, including scanner type, scan time, respiratory movements, reconstruction algorithms, voxel size, etc., may affect the repeatability of radiomics (69, 83).

There are two iterative reconstruction algorithms commonly used in PET/CT images: Ordered Subset Expectation Maximization (OSEM) and Block Sequential Regularized Expectation Maximization (BSREM). It seems that BSREM shows more sensitivity towards reconstruction. According to an evaluation by Schwyzer et al., AI performed significantly better on images with BSREM than OSEM (84). Another challenge is how to remove the image noise caused by the inherent characteristics of PET/CT equipment. Veenland et al. investigated the sensitivity of four different texture feature selection methods to image noise and blur, and found that the existence of noise reduced the discrimination performance of all texture features (85, 86).

Some features depend on different segmentation methods, which also affects the reproducibility and repeatability of radiomics. The traditional method is manual segmentation, which is undoubtedly time-consuming and labor-dependent, and variability between and within observers is inevitable (87). In recent years, automatic and semi-automatic segmentation techniques have been recommended to improve efficiency and reproducibility (88). Bi et al. proposed a recurrent fusion network (RFN) for automatic tumor segmentation on PET/CT imaging, which progressively fused the intermediary segmentation results from individual phases to minimize the risk of inconsistent feature learning. This result allows tumors with uneven texture to be segmented and consistent segmentation results can be achieved on various network backbones (26).

A prior article (89) evaluated studies in this field through the Transparent Reporting of a multivariable prediction model for Individual Prognosis or Diagnosis (TRIPOD) (90). Common problems included small datasets, a lack of clinically relevant comparators, independent testing, and standards for reporting the studies. Recently, a statement provided a consensus-based reporting guideline for the Developmental and Exploratory Clinical Investigations of Decision support systems driven by Artificial Intelligence (DECIDE-AI) (91). Unlike Tripod-AI, which focuses on specific research designs, Decision-AI is a reporting guideline for the evaluation phase for early, small-scale and real-time clinical evaluation of decision support systems based on AI. As we said, there are very few AI models that have been put into clinical application in the field of PET/CT radiomics, this report emphasizes that it is important to assess actual clinical performance at a small scale after the preclinical stage and before further large-scale clinical trials. Furthermore, the report emphasized the role of human in decision-making, and experts agreed that the variability of patients and users should be considered when evaluating artificial intelligence systems.

At present, no definitive conclusion can be drawn on which approach in radiomics and AI should be preferred. Compared with machine learning, deep learning has more advantages in tumor segmentation and processing big data sets, but its black box theory lacks explanation (92). At present, there is no universally accepted method to test trust in the context of clinical artificial intelligence, which can help explain why Decision-AI does not include interpretability and trust issues in its guidelines. In the future, joint strategic management is needed to improve the clinical translatability and practicability of radiomics and AI in order to gain the trust of users (93).

The disease management pattern of NSCLC has been greatly improved in the past few decades. The introduction of screening programs for high-risk groups has significantly improved the early diagnosis and survival rate of NSCLC, unfortunately, more than half of patients have locally advanced or metastatic diseases (stage III or IV) at the time of diagnosis (94). From this starting point, clinicians still face many difficulties. First, under the trend of personalized medicine, more accurate diagnosis and subtype classification of tumors are needed. It has been proved that immunohistochemical markers, such as thyroid transcription factor -1(TTF-1), Napsin A, CK5/6, P63 and P40, can help to improve the accuracy of histological diagnosis (95). However, as we have already mentioned, a single biopsy of a small specimen cannot reflect the whole tumor. By contrast, PET/CT radiomics has been considered to reflect intra-tumor heterogeneity. This is especially important for inoperable patients and large tumors that are inconvenient for histological evaluation of the whole tumor. On the basis of radiomics, radiogenomics is gradually emerging. Radiogenomics correlates a large number of radiomics features with genomic data to reveal the potential molecular mechanism, providing a promising means of non-invasive diagnosis at the molecular level (96).

With the successful introduction of molecular markers in the clinic, targeted therapy and immunotherapy have become important treatment options, but there are still some limitations in their development. There is no evidence of targeted therapy to cure non-small cell lung cancer. Patients treated with EGFR TKIs will develop drug resistance with long-term use, leading to disease progression (97). Immunotherapy may achieve a lasting response, but only a small number of selected patients benefit from it. In addition, subsequent immune-related adverse events (IrAEs), including checkpoint inhibitor pneumonia (CIP), have been widely reported (98). In this case, radiogenomics is expected to help effective treatment options and avoid expensive treatments that do not provide survival benefits, as well as predict treatment responses. In the articles we included in the analysis, there have been studies that simultaneously associated PET/CT radiomics with clinical outcomes and genomics. Mu et al. explored the alternative markers of PD-L1 expression based on PET/CT radiomics. The results revealed the biological driving force (hypoxia) behind deep learning and provided non-invasive decision for follow-up treatment (58). This is a direction of continuing efforts in the future, that is, radiogenomics data is expected to provide accurate diagnosis, tissue classification, treatment guidance and monitoring for NSCLC patients, so as to achieve more personalized patient care.

## Conclusion

Radiomics and artificial intelligence are considered as potential tools for the management of non-small cell lung cancer in the future, but the clinical transferability of results is an urgent problem to be solved. At present, there are several initiatives and recommendations to regulate the treatment and standardization of research reports. In the future, multi-center, prospective, larger cohort research is necessary. To overcome the technical repeatability and reproducibility defects, the establishment of a large multi-agency cooperative database covering a variety of imaging protocols and equipment, clinical environment, and patient characteristics may be the best way. Considering human factors, future developers should apply AI based on PET/CT radiomics analysis to more clinical trials to evaluate the applicability of computer-aided diagnosis systems in the local clinical environment. Furthermore, derived radiogenomics is expected to provide new insights into cancer biology, and there is still a long way to explore to achieve non-invasive decision-making under accurate medical care.

## Author contributions

QH was responsible for writing the manuscript and supplementing materials. KL and CY were responsible for proposing amendments and revising the manuscript. YW, RH, MG, and YX were responsible for reviewing the manuscript on the technical and clinical aspects. YH and LC was the overall guide and was responsible for the whole project. All authors contributed to the article and approved the submitted version.
